# Developing outcome, process and balancing measures for an emergency department longitudinal patient monitoring system using a modified Delphi

**DOI:** 10.1186/s12873-018-0220-3

**Published:** 2019-01-14

**Authors:** Marie E. Ward, Abel Wakai, Ronald McDowell, Fiona Boland, Eoin Coughlan, Moayed Hamza, John Browne, Ronan O’Sullivan, Una Geary, Fiona McDaid, Éidín Ní Shé, Frances J. Drummond, Conor Deasy, Eilish McAuliffe

**Affiliations:** 1 School of Nursing, Midwifery and Health Systems, C129, UCD Health Sciences Centre, University College Dublin, Belfield, Dublin, 4 Ireland; 2 Emergency Care Research Unit (ECRU), Division of Population Health Sciences (PHS), Royal College of Surgeons in Ireland (RCSI), Dublin 2 and Department of Emergency Medicine, Beaumont Hospital, Dublin, 9 Ireland; 3 General Practice and HRB Centre for Primary Care Research, Royal College of Surgeons in Ireland, Cancer Epidemiology and Health Services Research Group, Centre for Public Health, Queen’s University Belfast, Belfast, BT126BA UK; 4 Division of Population Health Sciences, Royal College of Surgeons in Ireland, Dublin, Ireland; 5 Department of Epidemiology and Public Health, University College Cork, Western Rd, Cork, Ireland; 6 Bon Secours Hospital, Cork, Ireland; 7 Department of Emergency Medicine, St James’s Hospital, Dublin, 8 Ireland; 8 Department of Emergency Medicine, Naas Hospital, Naas, Co, Kildare, Ireland; 9 Cancer Research@UCC, University College Cork, Cork, Ireland; 10 Department of Emergency Medicine, Cork University Hospital, Cork, Ireland

**Keywords:** Emergency department, Early warning score system, Longitudinal patient monitoring, Evaluation measures

## Abstract

**Background:**

Early warning score systems have been widely recommended for use to detect clinical deterioration in patients. The Irish National Emergency Medicine Programme has developed and piloted an emergency department specific early warning score system. The objective of this study was to develop a consensus among frontline healthcare staff, quality and safety staff and health systems researchers regarding evaluation measures for an early warning score system in the Emergency Department.

**Methods:**

Participatory action research including a modified Delphi consensus building technique with frontline hospital staff, quality and safety staff, health systems researchers, local and national emergency medicine stakeholders was the method employed in this study. In Stage One, a workshop was held with the participatory action research team including frontline hospital staff, quality and safety staff and health systems researchers to gather suggestions regarding the evaluation measures. In Stage Two, an electronic modified-Delphi study was undertaken with a panel consisting of the workshop participants, key local and national emergency medicine stakeholders. Descriptive statistics were used to summarise the characteristics of the panellists who completed the questionnaires in each round. The mean Likert rating, standard deviation and 95% bias-corrected bootstrapped confidence interval for each variable was calculated. Bonferroni corrections were applied to take account of multiple testing. Data were analysed using Stata 14.0 SE.

**Results:**

Using the Institute for Healthcare Improvement framework, 12 process, outcome and balancing metrics for measuring the effectiveness of an ED-specific early warning score system were developed.

**Conclusion:**

There are currently no published measures for evaluating the effectiveness of an ED early warning score system. It was possible in this study to develop a suite of evaluation measures using a modified Delphi consensus approach. Using the collective expertise of frontline hospital staff, quality and safety staff and health systems researchers to develop and categorise the initial set of potential measures was an innovative and unique element of this study.

**Electronic supplementary material:**

The online version of this article (10.1186/s12873-018-0220-3) contains supplementary material, which is available to authorized users.

## Background

Longitudinal patient monitoring systems (Early Warning Score (EWS) or Track and Trigger system (TTS)) have been widely recommended for use to detect clinical deterioration in patients [1]. The emergency department (ED) is a unique healthcare environment. ED patients are likely to be unknown to ED clinical staff and present with undifferentiated symptoms. They usually have to be managed with limited clinical information, through small windows of time and focus. Existing longitudinal patient monitoring systems developed for hospital inpatients may not be suitable for the ED [2, 3]. The early recognition of patient deterioration is also a key patient safety strategy for ED patients. It enables timely clinical intervention and transfer to a higher level of care in order to prevent adverse patient outcomes [4, 5]. A recent systematic review found that early warning systems ‘seem to predict adverse outcomes in adult patients of varying acuity presenting to the ED but there is a lack of high quality comparative studies to examine the effect of using early warning systems on patient outcomes’ [6]. There is also a lack of published measures for evaluating the effectiveness of longitudinal patient monitoring systems in the ED setting and the challenges of developing such measures have been outlined [7].

The Irish National Emergency Medicine Programme (EMP), aimed at improving the safety and quality of ED patient care, developed and piloted an ED-specific longitudinal patient monitoring system known as ED-ACE where ACE is an acronym for Adult Clinical Escalation. This study is part of a larger research programme, which saw the first full-scale iterative implementation of ED-ACE in a large urban acute hospital [7]. The main objective of this study was to develop a consensus among key stakeholders in the hospital frontline healthcare staff, quality and safety (Q&S) experts and health system researchers for outcome, process and balancing measures to measure the effectiveness of ED-ACE.

## Methods

### Methodological approach

The methodology to develop the evaluation measures was a two-stage process. Stage One consisted of a workshop with the research programme’s Participatory Action Research Group (PAR) group. All 13 members were invited to attend. Ten members attended (Table 1). The purpose of the workshop was to build on the collective professional experience of the members to gather suggestions regarding the evaluation measures to be used for the implementation of ED-ACE. Participants were informed about and asked to consider the Institute for Healthcare Improvement (IHI) framework for developing outcome, process and balancing measures [8]. The ‘stickies’ method was used to allow each individual to generate suggested outcome, process and balancing measures [9].

**Table 1:** Study Participants

PAR Workshop Participants	Delphi Participants
The 10 workshop participants included the following: Director Centre for Nurse Education; Assistant Director of Nursing; Consultant in Intensive Care; ED Business Manager; ED Clinical Nurse Manager; ED Clinical Nurse Facilitator; EM Consultant and Clinical Lead for the project; Professor of Health Systems and joint PI on project; Senior Research Fellow in Human Factors; Postdoctoral Researcher	Round 1: 22 nursing staff, 19 medical staff, 5 academics and researchers, 2 managers and 1 Health and Social Care Professional. Round 2: 12 nursing staff, 19 medical staff, 3 academics and researchers, 4 managers and 1 Health and Social Care Professional. Characteristics of Delphi panellists are outlined in Table 2.

In Stage Two, an electronic modified-Delphi study was undertaken to develop a consensus on a suite of measures to be used for the evaluation of ED-ACE [10]. The evaluation workshop was the modification on the Delphi and the rest of the process proceeded as a standard Delphi. A multidisciplinary Delphi panel was created to include the research team (including Health Systems, Epidemiology and Public Health, Patient Q&S, and Human Factors researchers); members of the project’s Scientific Advisory Group; Consultants, Registrars, Advance Nurse Practitioners (ANPs) from the ED at the planned implementation site who had not been involved in the research; all members of the EMP and the EMP’s Emergency Medicine Nursing Interest Group (ENIG); the Lead EM Consultant and EM nursing leads in all similar-sized EDs in Ireland. Fifty-eight professionals in total were invited to participate, 49 of which participated in Round 1 and 39 in Round 2 (Tables 1 and 2).

**Table 2:** Characteristics of Delphi panellists

		Round One	Round Two
	Number invited to participate	58	58
	Number of respondents	49 (84.5%)	39 (67.2%)
Professional background	Nursing	22 (44.9%)	12 (30.8%)
	Medical	19 (38.8%)	19 (48.7%)
	Academic /Faculty	3 (6.1%)	2 (5.1%)
	Managers	2 (4.1%)	4 (10.3%)
	Researchers	2 (4.1%)	1 (2.6%)
	Health and Social Care Professionals	1 (2.0%)	1 (2.6%)
Currently working in an ED	Yes	38 (77.5%)	31 (79.5%)
Based at hospital where ED-ACE implementation study being conducted	Yes	17 (34.7%)	16 (41.0%)

The Delphi approach was used in this study to reach consensus because other commonly used consensus group methods (for example, focus groups) were not cost-effective and feasible due to the fact that the panel for this study represented diverse geographical locations, making it impractical and costly to meet in person [11]. Other reasons for using the Delphi consensus approach for this study include participant anonymity (to each other, though not to the study’s lead researcher) and the avoidance of groupthink or domination that might arise in a face-to-face discussion [12]. Using the multi-disciplinary expertise of the PAR group to develop and categorise the initial set of potential measures was an innovative and unique element of this study.

The evaluation measures for the Delphi study were entered into SurveyMonkey software (https://www.surveymonkey.com/) to create an online/web-based electronic questionnaire that was used for both rounds of the Delphi study. A pilot study was then carried out based on three members of the research team assessing the content and flow of the draft questionnaire as well as ensuring the clarity of the measures and their categorisation. The feedback comments from the pilot study were used to create the final study questionnaire and an email containing the web link to the questionnaire was sent to all the Delphi panellists. This email also included a cover letter outlining the overall study objectives, how the initial list of measures was developed and an explanation of the Delphi process. Participation was on a voluntary basis and in keeping with the Delphi process participants were assured that their responses would be anonymous. Completion of the questionnaire was also taken as consent to participate. As the completion of the study questionnaire was anonymous, a background section called ‘Source of Expertise’ (Part A) was included in the questionnaire to capture background information (Please see Additional file 1: Appendix A for the Delphi R1 questionnaire).

### Data and statistical analysis

Descriptive statistics were used to summarise the characteristics of the panellists who completed the questionnaires in each round. The mean Likert rating, standard deviation and 95% bias-corrected bootstrapped confidence interval (CI) for each variable was calculated. Greatorex and Dexter [13] concluded that, although statistics such as the mean and standard deviation assume an interval scale, the mean can be understood to represent group opinion and the standard deviation the amount of disagreement within the panel. Missing data was due to dropout with some panellists failing to progress through consecutive sections of the questionnaire (see below) although where panellists ranked measures within a section they ranked all measures. All responses and rankings were included in the analyses.

Ensuring participant anonymity meant it was not possible to analyse at the individual (panellist) level change in relation to how measures, which were included in both rounds of the Delphi process, were ranked. However, where a variable reached the ‘high agreement’ threshold in R2 but not in R1, a one-sided test was used to determine whether the proportion of panellists who rated the variable as ‘Important’ or ‘Very Important’ in R2 was significantly higher than in R1. Bonferroni corrections were applied to take account of multiple testing. Data were analysed using Stata 14.0 SE.

## Results

### Stage one (workshop)

**Fig. 1:**
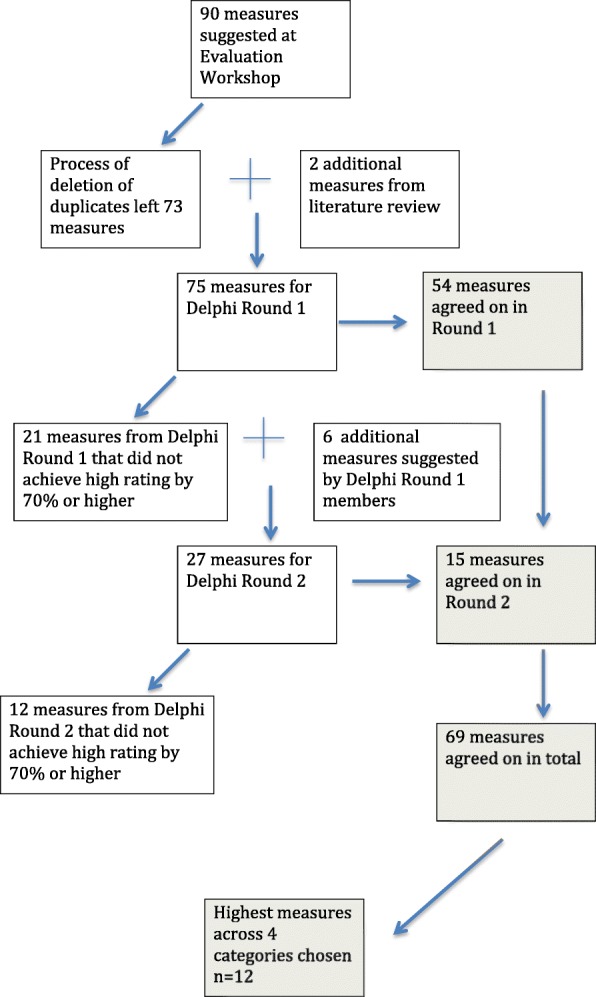
Flowchart of development of measures

An initial list of 90 potential measures was developed at the workshop. After duplicates were removed there were 73 potential measures remaining (Fig. 1). A scoping literature review was conducted to explore evaluation measures used in other studies of longitudinal patient monitoring systems being implemented in the ED setting. Two additional measures were added following the literature review.

Following the workshop, the list of proposed measures was categorised into outcome, process and balancing measures and more specifically into the following categories:

- Treatment process measures examining the treatment process of patients in the ED and how that might be affected by ED-ACE.
- Implementation process measures exploring the implementation, receipt and setting of implementing ED-ACE and help in the interpretation of the outcome results.
- Outcome Measures to determine if ED-ACE results in an improvement in patient outcomes.
- Balancing measures exploring the use of ED-ACE from different dimensions and the possible impact of its use on other areas of the ED and the wider hospital system.

### Stage two (Delphi consensus process)

The first round (R1) Delphi questionnaire contained 75 potential measures which participants were asked to rate on a 5-point Likert scale as very unimportant, unimportant, neither unimportant or important, important, very important for the evaluation of ED-ACE. This phase of the research was about determining the perceived importance of each measure by the key stakeholders. Assessing measures for feasibility and determining minimum data sets for measures would come later. At least 70% of Delphi panellists had to rate a potential measure in the ‘high agreement range’ (‘important’ and ‘very important’), for the measure to be selected for inclusion in the final suite of measures [11]. Participants were also invited to add any additional measures they felt should to be included in the Delphi round two (R2) questionnaire. The R1 questionnaire was emailed to 58 participants in August 2015. Personalised emails were sent to all the participants external to the project to improve the response rate. Two separate email reminders were sent after 3 weeks and then after one further week. The survey closed on 15th September 2015. Responses to R1 were analysed and proposed additional measures were collated (Fig. 1 provides a flowchart of the entire process). For R2 all 58 R1 participants were again invited to participate. This time they were asked to rate 27 potential measures. As in R1, personal email invitations to participate and reminders to the Delphi panellists were sent in R2.

### Characteristics of the panel

Table 2 details the characteristics of the Delphi panellists. The same group of 58 stakeholders were invited to participate in both rounds of the Delphi study. The response rates were 84.5% for R1 and 67.2% for R2. In R1 thirty-nine panellists rated the treatment process measures, thirty-five the implementation process measures, thirty-four the outcome measures and thirty-one the balancing measures. In R2 thirty-five panellists rated the treatment process measures, thirty-two the implementation process measures, and thirty-one both the outcome measures and the balancing measures.

### Stability of items ranked in both rounds

Twenty two variables, which did not meet the threshold for inclusion following R1, were revised if considered necessary for clarification, and panellists were asked to rate these again in R2 (in addition to other variables suggested by the Panellists in R1). 13 of these 22 variables were rated as ‘Important’ or ‘Very Important’ following R2 and these are listed in Table 3. However the proportion of panellists who ranked the variables as ‘Important’ or ‘Very Important’ in R2 was only significantly higher than in R1 for five of the variables. One of the 22 variables, “Number of patients who are in ED waiting for in-patient beds”, had the highest mean Likert score of all balancing measure variables across both rounds (4.33, 95%CI (3.98,4.63)).

**Table 3:** Variables from R1 attaining ‘high agreement’ in R2

Variable domain	Round One			Round Two			*p*-value
	No of respondents	Variable	No (%) panellists ranking variable Important/ Very important	No of respondents	Variable	No (%) panellists ranking variable Important/ Very important	
Treatment	39	Time of completion of admitting/consulting team assessment to time of ED departure	26 (66.7%)	35	Time of completion of admitting/consulting team assessment *in the ED* to time of ED departure	25 (71.4%)	0.66
Treatment	39	ICU admission rate	22 (56.4%)	35	ICU admission rate	30 (85.7%)	0.01
Implementation	35	ICU referral rate	23 (65.7%)	32	ICU referral rate	25 (78.1%)	0.78
Implementation	35	Resuscitation room activity level	22 (62.9%)	32	Resuscitation room activity level	30 (93.8%)	0.01
Implementation	35	Number of times ISBAR communication tool was used to communicate the need for escalation	22 (62.9%)	32	Number of times ISBAR communication tool was used to communicate the need for escalation	25 (78.1%)	0.52
Implementation	35	Health Professionals Work Index (HPWI) survey to measure autonomy and control over practice; work place relationships; managerial support and availability of resources	20 (57.1%)	32	*Staff perception of availability of support and resources*	27 (84.4%)	0.04
Implementation	35	Number of people being triaged	18 (51.4%)	32	Number of *patients* triaged	27 (84.4%)	0.01
Implementation	35	Minnesota Job Satisfaction Questionnaire to measure job satisfaction	16 (45.7%)	32	*Job satisfaction for ED staff*	30 (93.8%)	< 0.001
Treatment (Round 1) /utcome (Round 2)	39	Admission to Intensive Care Unit (ICU) within 2 days of having been assessed and treated and deemed appropriate for admission to a hospital ward from ED	25 (64.1%)	31	Admission to Intensive Care Unit (ICU) within 2 *h* of having been assessed and treated and deemed appropriate for admission to the ICU from the ED	22 (71.0%)	0.55
Outcome	34	In-hospital mortality rate	23 (67.6%)	31	In-hospital mortality rate	24 (77.4%)	0.38
Balancing	32	Service delivery measured through e.g. resources (beds, equipment etc.) against recommended requirements	22 (68.8%)	30	Service delivery measured through e.g. *available* resources (beds, equipment etc.) against recommended requirements	23 (76.7%)	0.72
Balancing	32	Average length of stay (AVLOS) in hospital for patients who come through ED	22 (68.8%)	30	Average length of stay (AVLOS) in hospital for patients who come through ED	23 (76.7%)	0.72
Balancing	32	Number of patients waiting for in-patient beds	21 (65.6%)	30	Number of patients *who are in the ED* waiting for in-patient beds	24 (80.0%)	0.30

### ED-ACE evaluation measures

A suite of 69 process (treatment and implementation process), outcome and balancing measures for evaluating the effectiveness of ED-ACE were developed (Fig. 1). Table 4 details the three measures in Delphi R1 and R2 with the highest mean Likert ratings per category thus leaving a set of the 12 highest-ranked measures. The purpose of using the Delphi technique was to reach consensus on which process, outcome and balancing measures would be the most important to evaluate ED-ACE. Table 4 therefore includes both those measures that reached consensus in R1, and therefore excluded from R2, as well as those reaching consensus in R2 across the four categories of process (treatment and implementation process), outcome and balancing measures [8]. Thus, while some clinical treatment variables may have rated higher than the top three balancing variables it was considered important to represent the top three variables across the four categories.Additional file 2 Appendix 2 provides a list of the mean Likert ratings for all of the proposed measures, the 69 that reached consensus as the most important and the remaining 34 that did not reach consensus.

**Table 4:** The proposed 12 measures with the highest mean ratings per IHI framework category

IHI Category	Highest Mean Rated Variable	Mean Rating (95% CI)
(A) Treatment Process Measures	Early detection and treatment of patients at risk of sepsis	4.95 [4.82, 4.97]
	Early identification and treatment of life-threatening complications	4.90 [4.82, 5.00]
	Early detection and treatment of patients with chest pain at risk of myocardial infarction	4.85 [4.74, 4.95]
(B) Implementation Process Measures	Number of patients who deteriorated as identified by ED-ACE	4.63 [4.43, 4.77]
	Number of patients whose care was escalated as a result of using ED-ACE	4.54 [4.20, 4.74]
	Number of re-triages that took place as a result of using D-ACE	4.31 [4.08, 4.49]
(C) Outcome Measures	Reduction in the number of serious incidents in the ED	4.59 [4.33, 4.77]
	Reduction in the number of unexpected deaths in the ED	4.41 [4.24. 4.62]
	Prevalence of deterioration in ED patients	4.38 [4.06, 4.68]
(D) Balancing Measures	Number of patients who are in the ED waiting for in-patient beds	4.33 [3.98, 4.63]
	Service delivery measured through actual staffing levels against recommended staffing levels	4.25 [3.97, 4.53]
	Staff adherence to treatment guidelines (e.g. treatment guidelines for acute stroke and acute myocardial infarction)	4.19 [3.88, 4.53]

## Discussion

The objective of this study was to reach a consensus regarding evaluation measures to measure the effectiveness of an ED-specific longitudinal patient monitoring system. While 69 measures reached consensus we would like to focus on the top- three ranked measures across the four IHI recommended categories for evaluating the implementation of a new Q&S initiative. The highest-ranked *treatment measures* in this study relate to life-threatening clinical conditions. This has face validity as the essence of the specialty of emergency medicine is to manage acute and urgent illness and injury. Early detection and treatment of patients at risk of sepsis is the highest-ranked measure in this study; this is not surprising given 60% of hospital deaths in Ireland have an infection or sepsis diagnosis and there has been considerable work done nationally and internationally on improving detection and treatment of sepsis [14]. Considering approximately 1 in 25 patients attending an Irish ED may have sepsis [15] and 1 in 100 patients attending an Irish ED may have severe sepsis or septic shock [16], this measure has high clinical relevance. Chest pain accounts for 5–20% of all ED admissions [17]. Causes of chest pain range from the benign (e.g., musculoskeletal chest pain) to potentially life-threatening conditions (e.g., acute coronary syndrome). The early detection and treatment of patients with chest pain at risk of myocardial infarction was rated highly in this study.

The highest-ranked *implementation process measures* in this study were the number of patients who deteriorated, whose care was escalated, and who were re-triaged as a result of using ED-ACE. The highest-ranked *outcome measures* related to the reduction in the number of serious incidents and unexpected deaths in the ED and prevalence of deterioration in ED patients. These outcome measures are consistent with the underlying principle of longitudinal patient monitoring systems, to detect and prevent patient deterioration [1].

Balancing measures are meant to detect any unintended consequences of implementing a new intervention [18]. The highest-ranked *balancing measures* related to the number of patients who are in the ED waiting for in-patient beds. This may reflect concerns about the possibility of worsening chronic crowding in most EDs if use of ED-ACE resulted in more patients being transferred from the ED waiting room to the already crowded clinical care areas within the ED. The second highest ranked balancing measure was service delivery as measured by actual staffing levels against recommended staffing levels. Considering ED staffing is the single most important factor in providing a high quality, timely and clinically effective service to patients [19], this may reflect concerns about the ED staffing resource needed to carry out regular monitoring of ED patients. The third balancing measure chosen, staff adherence to treatment guidelines (e.g., for acute stroke), may also reflect concerns about the adequacy of ED staffing resource to implement ED-ACE while concomitantly delivering patient care consistent with existing clinical guideline recommendations. Given resource limitations and ED crowding it is possible that shortcuts may be taken in implementing all components of ED-ACE. This is something that would need to be monitored during implementation.

### Limitations

Firstly, valid and reliable measures depend on the availability of high quality data [20] and while this study is the first step in developing consensus on a suite of measures, a separate study is required to determine how feasible and cost-effective it will be to collect the minimum data set required for implementing the measures [21]. Secondly, while we considered the views of frontline staff and researchers, there was no patient representation in the consensus development process. Thirdly, while the response rate to R1 was high (84.5%) this did drop to 67.2% in R2. In order to maintain the rigour of this technique, a response rate of 70% is suggested, but as Hasson et al. [22] note to achieve this, the researcher must know the identity of respondents, and non-respondents must be pursued individually. Given that our study was anonymous and we could not pursue participants individually a response rate of 67.2% can be considered acceptable. Also given that our study was anonymous we were unable to examine changes within individual respondents between rounds. Finally, the study investigators assumed that all the Delphi panellists had relevant knowledge regarding the evidence base for all the measures that reached consensus. However, it is conceivable that not all the panellists were aware of the evidence supporting all the measures that reached agreement.

## Conclusion

The Delphi technique has been used previously to develop quality of clinical care indicators for EDs [20]. In this study it has proved effective in contributing to the development of a suite of 12 treatment and implementation process, outcome and balancing measures for measuring the effectiveness of an ED-specific longitudinal patient monitoring system. In our knowledge, this is the first study to develop a suite of measures to evaluate the effectiveness of an ED-specific longitudinal patient monitoring system. While acknowledging the limitations outlined above, we consider this study a necessary starting point for the development of valid and reliable measures to evaluate the effectiveness of ED-ACE.

## Additional files

Additional file 1: Appendix 1: Delphi Instrument Round 1 This contains the full delphi questionnaire for Round 1 of the study (PDF 240 kb)

Additional file 2: Appendix 2: Delphi Measures Mean Likert Scores This provides a list of the mean Likert ratings for all of the proposed measures, the 69 that reached consensus as the most important and the remaining 34 that did not reach consensus. (DOCX 39 kb)

## Abbreviations

ANP: Advance nurse practitioners
CI: Confidence interval
ED: Emergency department
ED-ACE: Emergency department adult clinical escalation
EMP: Emergency medicine programme
ENIG: Emergency medicine nursing interest group
EWS: Early warning score
IHI: Institute for healthcare improvement
PAR: Participatory action research group
Q&S: Quality and safety
TTS: Track and trigger system
