# Supplementary material for: Developing outcome, process and balancing measures for an emergency department longitudinal patient monitoring system using a modified Delphi
Source: BMC Emerg Med. 2019 Jan 14;19:7. doi: 10.1186/s12873-018-0220-3 (PMC6332627; doi:10.1186/s12873-018-0220-3)
Supplement: Supplementary file 1 — Appendix 1: Delphi Instrument Round 1 This contains the full delphi questionnaire for Round 1 of the study (PDF 240 kb) [file 12873_2018_220_MOESM1_ESM.pdf]

Introduction

An ED-specific longitudinal patient monitoring system (known as ED-ACE) has been designed for the purpose of detecting deterioration in a patient's condition in a timely manner and assisting in the decision and process of escalating care where appropriate. In order to test the effectiveness of this system, there must first be consensus on what variables should be measured to reliably determine its effectiveness. The variables outlined in the questionnaire have been generated through a brainstorming session held with ED staff and researchers involved in implementing the system followed by a review of the literature.

The purpose of this Modified Electronic Delphi Technique is to utilise the expertise of a representative cross-section of key stakeholders to reach consensus on the measures (from those listed and in Round 2 from any additional measures that stakeholders might suggest here) that are most important in determining the effectiveness of the longitudinal patient monitoring system. This survey has 5 parts:

Part A: Source of Expertise

Part B: Treatment Process Measures – these measures examine the treatment process of patients in the ED and how that might be affected by ED-ACE

Part C: Implementation Process Measures – these measures examine the implementation of ED-ACE, its use by staff and its impact on staff.

Part D: Outcome Measures – these measures should determine if ED-ACE results in improvement in patient outcomes.

Part E: Balancing Measures – these measures should determine the impact of ED-ACE beyond its specific impact on ED patients.

We would be grateful if you would please answer all questions and rate all the measures.

Part A: Sources of Expertise

* Please indicate the staff category to which you belong:

- ○ Nurse
- ○ Doctor
- ○ Health & Social Care Professional (formerly AHP)
- ○ Administration
- ○ Management
- ○ Academic
- ○ Researcher

Other (please specify)

[ ]

* Do you currently work in an Emergency Department?

- ○ Yes
- ○ No

If no, have you previously worked in an Emergency Department?

- ○ Yes
- ○ No

* Are you currently working in Cork University Hospital?

- ○ Yes
- ○ No

* Are you a member of the RED-ACE research team or did you participate in the initial brainstorming session to develop measures?

- ○ Yes
- ○ No

Part B: Treatment Process Measures

Treatment Process Measures – these measures examine the treatment process of patients in the ED and how that might be affected by ED-ACE.

Please read the measures below, and then indicate the degree of your agreement or disagreement with how important the measure is to understanding the critical factors to successful implementation of the ED-ACE by ticking one of the following options:

1 = very unimportant, 2 = unimportant, 3 = neither important nor unimportant, 4 = important, 5 = very

important

If you wish you may suggest another measure ('other') that you feel is so important we should add it to the list for the next Delphi.

* Treatment Process Measures

	Very Unimportant	Unimportant	Neither Important nor Unimportant	Important	Very important
Early detection and treatment of patients at risk of Sepsis	○	○	○	○	○
Early detection and treatment of patients at risk of Diabetic Keto Acidosis (DKA)	○	○	○	○	○
Early detection and treatment of patients with Head injury (HI)	○	○	○	○	○
Early detection and treatment of patients at risk of Stroke	○	○	○	○	○
Early detection and treatment of patients with Chest pain at risk of Myocardial Infarction (MI)	○	○	○	○	○
Early detection of cerebral vascular events and definite treatment	○	○	○	○	○
Early identification and treatment of life threatening complications	○	○	○	○	○
Reduction in the number of unplanned patient re-attendance	○	○	○	○	○
Reduction in the number of Left without being seen (LWBS) patients	○	○	○	○	○
Reduction in time to transfer from waiting room to appropriate ED treatment area	○	○	○	○	○
Time to be seen by hospital in-house specialist following escalation	○	○	○	○	○

	Very Unimportant	Unimportant	Neither Important nor Unimportant	Important	Very important
Time to be seen by senior ED clinician following escalation	○	○	○	○	○
Time to receive sepsis bundle/ antibiotic for the deteriorating patient	○	○	○	○	○
Time to intervention following escalation	○	○	○	○	○
Time to diagnostics following escalation	○	○	○	○	○
Time to transfer to ICU where ICU admission deemed necessary	○	○	○	○	○
Ambulance arrival time to triage time	○	○	○	○	○
ED arrival time to triage time	○	○	○	○	○
Triage time to time seen by treating clinician	○	○	○	○	○
Time seen by treating clinician to time of disposition decision	○	○	○	○	○
Time of disposition decision to time seen by admitting / consulting team	○	○	○	○	○
Time seen by admitting / consulting team to time of completion of their assessment	○	○	○	○	○
Time of completion of admitting / consulting team assessment to time bed requested on Patient Administration System (PAS)	○	○	○	○	○
Time of completion of admitting / consulting team assessment to time of ED departure	○	○	○	○	○

	Very Unimportant	Unimportant	Neither Important nor Unimportant	Important	Very important
Admission to Intensive Care Unit (ICU) within 2 days of having been assessed and treated and deemed appropriate for admission to a hospital ward from ED	○	○	○	○	○
Number of critical care reviews	○	○	○	○	○
ICU admission rate	○	○	○	○	○
Coronary Care Unit (CCU) admission rate	○	○	○	○	○
Clinical Decisions Unit (CDU) admission rate	○	○	○	○	○
Unanticipated ICU admission rate	○	○	○	○	○

Other (please specify)

Part C: Implementation Process Measures

Implementation Process Measures – these measures examine the implementation of ED-ACE, its use by staff and its impact on staff. Implementation process measures explore the implementation, receipt and setting of implementing ED-ACE and help in the interpretation of the outcome results.

They can help distinguish between interventions that are inherently faulty (failure of intervention concept or theory) and those that are badly delivered (implementation failure).

Please read the measures below, and then indicate the degree of your agreement or disagreement with how important the measure is to understanding the critical factors to successful implementation of the ED-ACE by ticking one of the following options:

1 = very unimportant, 2 = unimportant, 3 = neither important nor unimportant, 4 = important, 5 = very important

If you wish you may suggest another measure ('other') that you feel is so important we should add it to the list for the next Delphi.

* Implementation Process Measures

	Very Unimportant	Unimportant	Neither Important nor Unimportant	Important	Very important
Staff survey on perceived usefulness of ED-ACE	○	○	○	○	○
Health Professionals Work Index (HPWI) survey to measure autonomy and control over practice; work place relationships; managerial support and availability of resources	○	○	○	○	○
Minnesota Job Satisfaction Questionnaire to measure job satisfaction	○	○	○	○	○
Survey of junior staff on their competence and confidence in using ED-ACE	○	○	○	○	○
Change in number of interruptions for senior staff	○	○	○	○	○
Time taken to train staff in ED-ACE	○	○	○	○	○
Number of patient self alerts of deterioration	○	○	○	○	○
Number of relative or other person alert to deterioration of person in waiting room	○	○	○	○	○
Number of times ED-ACE actually used to monitor patients post-triage vs number of times recommended to be used	○	○	○	○	○
Number of times ED-ACE actually used to monitor patients post-triage vs number of times recommended to be used by staffing levels	○	○	○	○	○

	Very Unimportant	Unimportant	Neither Important nor Unimportant	Important	Very important
Number of times ED-ACE actually used to monitor patients in cubicles vs number of times recommended to be used	○	○	○	○	○
Number of times ED-ACE actually used to monitor patients in cubicles vs number of times recommended to be used by staffing levels	○	○	○	○	○
Percentage compliance to use of ED-ACE against the number of patient presentations to ED	○	○	○	○	○
Number of patients who deteriorated as identified by ED-ACE	○	○	○	○	○
Number of patients whose care was escalated as a result of using ED-ACE	○	○	○	○	○
Number of times ISBAR communication tool was used to communicate need for escalation	○	○	○	○	○
Number of re-triages that took place as a result of using ED-ACE	○	○	○	○	○
Number of changes in triage category that took place as a result of using ED-ACE	○	○	○	○	○
Resuscitation Room activity level	○	○	○	○	○
Number of people being triaged	○	○	○	○	○
ICU referral rate	○	○	○	○	○
Ambulance turnaround time	○	○	○	○	○
Time taken to perform ambulance clinical handover	○	○	○	○	○

Other (please specify)

Part D: Outcome Measures

Outcome Measures – these measures should determine if ED-ACE results in improvement in patient outcomes.

Please read the measures below, and then indicate the degree of your agreement or disagreement with how important the measure is to determining the effectiveness of the ED-ACE by ticking one of the following options:

1 = very unimportant, 2 = unimportant, 3 = neither important nor unimportant, 4 = important, 5 = very important

If you wish you may suggest another measure ('other') that you feel is so important we should add it to the list for the next Delphi.

Please note the following definitions of incident and serious incident:
Incident is defined as "An event or circumstance which could have, or did lead to unintended and/or unnecessary harm. Incidents include adverse events which result in harm; near-misses which could have resulted in harm, but did not cause harm, either by chance or timely intervention; and staff or service user complaints which are associated with harm" (HSE, 2014 p.5). Harm is defined as "any physical or psychological injury or damage to the health of a person, including both temporary and permanent injury" (p.4).

Serious incident is defined as one "that results in death or serious harm" (HSE, 2014 p. 5) where serious harm is defined as "serious injury to a person, or serious damage done to a thing. An injury which creates a substantial risk of death or which causes serious disfigurement or substantial loss or impairment of the mobility of the body as a whole or of the function of any particular bodily member or organ" (p.5).

* Outcome measures

	Very Unimportant	Unimportant	Neither Important nor Unimportant	Important	Very important
Mortality rate of all patients treated in ED followed up at 7 days	○	○	○	○	○
Mortality rate of all patients treated in ED followed up at 30 days	○	○	○	○	○
In-hospital mortality rate	○	○	○	○	○
30 day outcome of patients whose care was escalated through use of ED-ACE	○	○	○	○	○
Reduction in the number of Unexpected deaths in the ED	○	○	○	○	○
Reduction in the number of Cardiac arrests in the ED	○	○	○	○	○
Reduction in the number of *incidents* in the ED (please see above for definition of incident)	○	○	○	○	○
Reduction in the number of *serious incidents* in the ED (please see above for definition of serious incident)	○	○	○	○	○
Effective management of pain for patients in the waiting room	○	○	○	○	○
Prevalence of deterioration in ED patients	○	○	○	○	○

Other (please specify)

Part E: Balancing Measures

Balancing Measures – these measures should determine the impact of ED-ACE beyond its specific impact on ED patients. Balancing measures look at a system from different directions/dimensions. They can help us answer questions like are changes designed to improve one part of the system causing new problems in other parts of the system?

Please read the Measures below, and then indicate the degree of your agreement or disagreement

with how important the Measure is to determining the impact of the using ED-ACE on service delivery by ticking one of the following options:

1 = very unimportant, 2 = unimportant, 3 = neither important nor unimportant, 4 = important, 5 = very important

If you wish you may suggest another measure ('other') that you feel is so important we should add it to the list for the next Delphi.

* Balancing Measures

	Very Unimportant	Unimportant	Neither Important nor Unimportant	Important	Very Important
Staff survey on patient safety culture	○	○	○	○	○
Service delivery measured through e.g. staffing levels against recommended requirements	○	○	○	○	○
Service delivery measured through e.g. resources (beds, equipment etc) against recommended requirements	○	○	○	○	○
Service delivery measured through e.g. meeting patient experience time (PET) in the ED of six hours for 95% of patients and nine hours for 100% of patients	○	○	○	○	○
Service delivery measured through e.g. number of Complaints received to ED	○	○	○	○	○
Service delivery measured through e.g. survey of Patient and Relative satisfaction with ED journey	○	○	○	○	○
Staff workload measured through e.g. observation and measurement of staff task completion times	○	○	○	○	○

	Very Unimportant	Unimportant	Neither Important nor Unimportant	Important	Very Important
Staff workload measured through e.g. survey on staff perceptions of workload balance	○	○	○	○	○
PET through the ED broken down into different stages of ED patient journey	○	○	○	○	○
Staff adherence to treatment guidelines for e.g. stroke and Myocardial Infarction (MI)	○	○	○	○	○
Number of patients waiting for in-patient beds	○	○	○	○	○
Average length of stay (AVLOS) in hospital for patients who come through ED	○	○	○	○	○

Other (please specify)

Thank you

Thank you very much for taking the time to completing Round 1 of this Modified Electronic Delphi Technique. We appreciate the time and effort that you have given to this and will be in touch within the next few weeks in relation to Round 2.
