# Supplementary material for: Developing outcome, process and balancing measures for an emergency department longitudinal patient monitoring system using a modified Delphi
Source: BMC Emerg Med. 2019 Jan 14;19:7. doi: 10.1186/s12873-018-0220-3 (PMC6332627; doi:10.1186/s12873-018-0220-3)
Supplement: Supplementary file 2 — Appendix 2: Delphi Measures Mean Likert Scores This provides a list of the mean Likert ratings for all of the proposed measures, the 69 that reached consensus as the most important and the remaining 34 that did not reach consensus. (DOCX 39 kb) [file 12873_2018_220_MOESM2_ESM.docx]

**Appendix 2: Variables selected from the Delphi process, ranked by mean Likert score**

| **Process Measure Domain** | **Delphi**  **Round** | **Variable** | **Mean Likert score**  **(95% CI)** |
| --- | --- | --- | --- |
| Treatment | 1 | Early detection and treatment of patients at risk of sepsis | 4.95 (4.82,4.97) |
| Treatment | 1 | Early identification and treatment of life-threatening complications | 4.90 (4.82,5.00) |
| Treatment | 1 | Early detection and treatment of patients with chest pain at risk of myocardial infarction (MI) | 4.85 (4.74,4.95) |
| Treatment | 1 | Time to receive sepsis bundle/antibiotic for the deteriorating patient with suspected or presumed sepsis | 4.82 (4.72,4.95) |
| Treatment | 1 | Early detection of cerebrovascular events and definitive treatment | 4.74 (4.51,4.84) |
| Treatment | 1 | Early detection and treatment of patients at risk of diabetic ketoacidosis (DKA) | 4.72 (4.54,4.85) |
| Treatment | 1 | Early detection and treatment of patients with head injury (HI) | 4.69 (4.56,4.85) |
| Implementation | 1 | Number of patients who deteriorated as identified by ED-ACE | 4.63 (4.43,4.77) |
| Outcome | 1 | Reduction in the number of serious incidents in the ED | 4.59 (4.33,4.77) |
| Treatment | 1 | Early detection and treatment of patients at risk of stroke | 4.56 (4.26,4.77) |
| Treatment | 1 | Time to intervention following escalation | 4.54 (4.31,4.69) |
| Implementation | 1 | Number of patients whose case was escalated as a result of using ED-ACE | 4.54 (4.20,4.74) |
| Treatment | 1 | Time to transfer to ICU where ICU admission deemed necessary | 4.51 (4.26,4.69) |
| Treatment | 1 | Time to be seen by a senior ED clinician following escalation | 4.44 (4.28,4.64) |
| Outcome | 1 | Reduction in the number of unexpected deaths in the ED | 4.41 (4.24,4.62) |
| Outcome | 1 | Prevalence of deterioration in ED patients | 4.38 (4.06,4.68) |
| Outcome | 1 | Reduction in the number of incidents in the ED | 4.35 (4.18,4.59) |
| Outcome | 1 | Effective management of pain for patients in the ED waiting room | 4.33 (4.09,4.56) |
| Balancing | 2 | Number of patients who are in ED waiting for in-patient beds | 4.33 (3.98,4.63) |
| Implementation | 1 | Number of re-triages that took place as a result of using ED-ACE | 4.31 (4.08,4.49) |
| Implementation | 1 | Number of changes in triage category that took place as a result of using ED-ACE | 4.31 (4.06,4.51) |
| Implementation | 1 | Resuscitation room activity level | 4.31 (4.09,4.56) |
| Treatment | 1 | Time to diagnostics following escalation | 4.26 (4.05,4.51) |
| Outcome | 1 | Reduction in the number of cardiac arrests in the ED | 4.26 (3.91,4.59) |
| Implementation | 2 | Job satisfaction of ED staff | 4.25 (4.00,4.50) |
| Balancing | 1 | Service delivery measured through e.g. staffing levels against recommended requirements | 4.25 (3.97,4.53) |
| Outcome | 1 | 30 day outcome of patients whose care was escalated through use of ED-ACE | 4.24 (3.79,4.53) |
| Outcome | 2 | Number of patients who are admitted to a hospital ward from the ED and subsequently deteriorate and are transferred to an ICU within 48 hours of departing the ED | 4.23 (4.00,4.52) |
| **Process Measure**  **Domain** | **Delphi**  **Round** | **Variable** | **Mean Likert score**  **(95% CI)** |
| Treatment | 1 | Triage time to time seen by treating clinician | 4.21 (4.05,4.44) |
| Balancing | 1 | Staff adherence to treatment guidelines for e.g. stroke and Myocardial Infarction (MI) | 4.19 (3.88,4.53) |
| Implementation | 1 | Number of times ED-ACE actually used to monitor patients in cubicles vs number of times recommended to be used | 4.14 (3.80,4.46) |
| Implementation | 1 | Number of times ED-ACE actually used to monitor patients post-triage vs number of times recommended to be used | 4.14 (3.86,4.46) |
| Implementation | 2 | Number of patients re-triaged | 4.13 (3.94,4.40) |
| Implementation | 1 | Staff survey on perceived usefulness of ED-ACE | 4.11 (3.82,4.31) |
| Treatment | 1 | Time to be seen by hospital in-house specialist following escalation | 4.10 (3.79,4.31) |
| Implementation | 1 | Change in number of interruptions for senior staff | 4.09 (3.74,4.34) |
| Implementation | 2 | Staff perception of availability of support and resources | 4.09 (3.84,4.41) |
| Balancing | 1 | Staff survey on patient safety culture | 4.09 (3.84,4.41) |
| Balancing | 1 | Service delivery measured through e.g. meeting patient experience time (PET) in ED of six hours for 95% of patients and nine hours for 100% of patients | 4.09 (3.81,4.38) |
| Implementation | 1 | Number of times ED-ACE actually used to monitor patients in cubicles vs number of times recommended to be used by staffing levels | 4.06 (3.66,4.40) |
| Outcome | 2 | Reduction in length of ICU/HDU admission | 4.06 (3.77,4.39) |
| Outcome | 2 | Admission to Intensive Care Unit (ICU) within 2 hours of having been assessed and treated and deemed appropriate for admission to the ICU from the ED | 4.06 (3.71,4.45) |
| Treatment | 1 | Reduction in time to transfer from waiting room to appropriate ED treatment area | 4.03 (3.77,4.28) |
| Treatment | 1 | Unanticipated ICU admission rate | 4.03 (3.79,4.28) |
| Implementation | 1 | Number of times ED-ACE actually used to monitor patients post-triage vs number of times recommended to be used by staffing levels | 4.03 (3.60,4.31) |
| Implementation | 1 | Survey of junior staff on their competence and confidence in using ED-ACE | 4.03 (3.71,4.29) |
| Outcome | 1 | Mortality rate of all patients treated in ED followed up at 7 days | 4.03 (3.65,4.32) |
| Balancing | 2 | Service delivery measured through e.g. available resources (beds, equipment etc.) against recommended requirements | 4.03 (3.73,4.37) |
| Balancing | 1 | Staff workload measured through e.g. observation and measurement of staff task completion times | 4.00 (3.75,4.25) |
| Implementation | 1 | Percentage compliance to use of ED-ACE against the number of patient presentations to ED | 3.97 (3.57,4.23) |
| Balancing | 1 | Service delivery measured through e.g. survey of patient and relative satisfaction with ED journey | 3.97 (3.75,4.25) |
| Implementation | 1 | Time taken to train staff in ED-ACE | 3.97 (3.69,4.14) |
| Implementation | 1 | Number of relative or other person alert to deterioration of person in ED waiting room | 3.94 (3.63,4.23) |
| Outcome | 1 | Mortality rate of all patients treated in ED followed up at 30 days | 3.94 (3.59,4.26) |
| Outcome | 2 | In-hospital mortality rate | 3.94 (3.55,4.22) |
| Implementation | 2 | Number of times ISBAR communication tool was used to communicate the need for escalation | 3.93 (3.59,4.28) |
| **Process Measure**  **Domain** | **Delphi**  **Round** | **Variable** | **Mean Likert score**  **(95% CI)** |
| Treatment | 2 | ICU admission rate | 3.91 (3.66,4.17) |
| Balancing | 1 | Staff workload measured through e.g. survey on staff perceptions of workload balance | 3.91 (3.63,4.19) |
| Implementation | 1 | Number of patient self alerts of deterioration | 3.91 (3.60,4.20) |
| Balancing | 2 | Average length of stay (AVLOS) in hospital for patients who come through ED | 3.90 (3.53,4.17) |
| Treatment | 1 | Time of patient disposition decision to time seen by admitting/consulting team | 3.90 (3.59,4.10) |
| Treatment | 1 | ED arrival time to triage time | 3.90 (3.54,4.18) |
| Treatment | 2 | Time of completion of admitting/consulting team assessment in the ED to time of ED departure | 3.89 (3.46,4.20) |
| Implementation | 2 | ICU referral rate | 3.88 (3.55,4.19) |
| Balancing | 1 | PET through the ED broken down into different stages of ED patient journey | 3.88 (3.50,4.22) |
| Treatment | 1 | Reduction in the number of left without being seen (LWBS) patients | 3.82 (3.59,4.05) |
| Balancing | 1 | Service delivery measured through e.g. number of complaints received to ED | 3.81 (3.63,4.06) |
| Treatment | 1 | Time seen by treating clinician to time of deposition decision | 3.77 (3.49,3.97) |
| Treatment | 1 | Reduction in the number of unplanned patient re-attendance | 3.62 (3.36,3.92) |
